# KomaMRI.jl: An open‐source framework for general MRI simulations with GPU acceleration

**DOI:** 10.1002/mrm.29635

**Published:** 2023-03-06

**Authors:** Carlos Castillo‐Passi, Ronal Coronado, Gabriel Varela‐Mattatall, Carlos Alberola‐López, René Botnar, Pablo Irarrazaval

**Affiliations:** ^1^ School of Biomedical Engineering and Imaging Sciences King's College London London UK; ^2^ Institute for Biological and Medical Engineering Pontificia Universidad Católica de Chile Santiago Chile; ^3^ Millennium Institute for Intelligent Healthcare Engineering (iHEALTH) Pontificia Universidad Católica de Chile Santiago Chile; ^4^ Electrical Engineering Pontificia Universidad Católica de Chile Santiago Chile; ^5^ Centre for Functional and Metabolic Mapping (CFMM), Robarts Research Institute Western University London Ontario Canada; ^6^ Department of Medical Biophysics, Schulich School of Medicine and Dentistry Western University London Ontario Canada; ^7^ Laboratorio de Procesado de Imagen Universidad de Valladolid Valladolid Spain

**Keywords:** Bloch equations, GPU, GUI, Julia, open source, simulation

## Abstract

**Purpose:**

To develop an open‐source, high‐performance, easy‐to‐use, extensible, cross‐platform, and general MRI simulation framework (Koma).

**Methods:**

Koma was developed using the Julia programming language. Like other MRI simulators, it solves the Bloch equations with CPU and GPU parallelization. The inputs are the scanner parameters, the phantom, and the pulse sequence that is Pulseq‐compatible. The raw data is stored in the ISMRMRD format. For the reconstruction, MRIReco.jl is used. A graphical user interface utilizing web technologies was also designed. Two types of experiments were performed: one to compare the quality of the results and the execution speed, and the second to compare its usability. Finally, the use of Koma in quantitative imaging was demonstrated by simulating Magnetic Resonance Fingerprinting (MRF) acquisitions.

**Results:**

Koma was compared to two well‐known open‐source MRI simulators, JEMRIS and MRiLab. Highly accurate results (with mean absolute differences below 0.1% compared to JEMRIS) and better GPU performance than MRiLab were demonstrated. In an experiment with students, Koma was proved to be easy to use, eight times faster on personal computers than JEMRIS, and 65% of test subjects recommended it. The potential for designing acquisition and reconstruction techniques was also shown through the simulation of MRF acquisitions, with conclusions that agree with the literature.

**Conclusions:**

Koma's speed and flexibility have the potential to make simulations more accessible for education and research. Koma is expected to be used for designing and testing novel pulse sequences before implementing them in the scanner with Pulseq files, and for creating synthetic data to train machine learning models.

## Abstract 2

Click here for author‐reader discussions

## 1 INTRODUCTION

Numerical simulations are an important tool for analyzing and developing new acquisition and reconstruction methods in MRI. Simulations allow us to isolate and study phenomena by removing unwanted effects, such as hardware imperfections, off‐resonance, and others. Additionally, with the increasing use of Machine Learning models, simulation becomes even more relevant, because it can be used to generate synthetic data for training,^1^, ^2^ or to construct signal dictionaries to infer quantitative measurements from the acquired data.^3^, ^4^ Moreover, simulations are an excellent tool for education and training, as hands‐on experience is a great way to assimilate the theoretical and practical components of MRI.^5^, ^6^, ^7^

MRI simulators can be application‐specific or general. Application‐specific simulators are efficient computationally and only consider a few relevant effects (e.g., POSSUM,^8^, ^9^ CAMINO,^10^ and others^11^, ^12^, ^13^). A common simplification is that the acquired signal is equal to the two‐/three‐dimensional Fourier transform of the image, not taking into account relaxation during the acquisition, and other elements of MRI physics. On the other hand, general simulators solve the Bloch equations making them computationally intensive but usable in a wider range of applications.

Currently one of the most used general MRI simulators is JEMRIS.^14^ This open‐source simulator considers many properties of interest in MRI, such as $M_{0}$, $T_{1}$, $T_{2}$, $T_{2}^{*}$, $\Delta B_{0}$, movement, etc. JEMRIS only uses CPU multithreading. Other alternatives are two closed‐source simulators: MRISIMUL^15^ and BlochSolver.^16^, ^17^ Both are accelerated through the use of GPUs. Originally, MRISIMUL did not have a Graphical User Interface (GUI), but recently a cloud‐based implementation called coreMRI has become available.^18^ In 2017 a new open‐source and GPU‐accelerated simulator was introduced: MRiLab.^19^ The main drawbacks of MRiLab are that it does not use self‐contained sequence files like JEMRIS, and that it was not designed to be extensible.

All of the previously mentioned simulators are written in C++ for speed. In practice, this may raise the bar for researchers in order to implement or modify these simulators.

The current open‐source alternatives use MATLAB‐based GUIs, resulting in nonintuitive interfaces. Furthermore, they do not support all Operating Systems (Table 1).

**TABLE 1:** Overview of general MRI simulators.

Name	GUI	GPU	Open	Cross‐platform
JEMRIS^14^	✓	×	✓	×
MRISIMUL^15^	×	✓	×	×
BlochSolver^16^	✓	✓	×	✓
MRiLab^19^	✓	✓	✓	×
coreMRI^15^, ^18^	✓	✓	×	✓

We believe that an ideal simulator should be general, fast, easy to use, extensible, open‐source, and cross‐platform. In this work, we developed an MRI simulation framework built from the ground up to satisfy these requirements.

To achieve these goals we made four important design decisions: programming language, compatibility with accepted standards, interface, and simulation method.

We chose the Julia programming language^20^ because its syntax is similar to MATLAB (widely used by the MRI community), its excellent GPU support,^21^, ^22^ and its speed is comparable to C/C++ (Julia is a compiled language). This has been shown to be the case in other MRI applications such as image reconstruction with MRIReco.jl,^23^ where the authors achieved speeds on par with state‐of‐the‐art toolboxes.^24^ In contrast to many other languages Julia can select the definition of a function to call at runtime via multiple dispatch. This is perhaps its most powerful feature of Julia. This allowed us to use syntax that more closely follows mathematical notation.

The inputs to our simulation framework are the scanner parameters, the phantom, and the pulse sequence. For the latter we offer the possibility to program it directly in the code or alternatively to read it from a file in the standard Pulseq format.^25^, ^26^, ^27^ The output raw data is stored in the standard ISMRMRD format.^28^ For reconstruction, our framework offers the possibility to use MRIReco.jl,^23^ any other reconstruction application that can read ISMRMRD, or direct programming of the code.

Using web technologies, we designed a GUI to improve accessibility for nonprogrammers and to facilitate the exploration of data and parameter tuning in a clear manner. This GUI also allows reading or writing the intermediate results.

We chose not to use an Ordinary Differential solver, like DifferentialEquations.jl, but to handcraft an MRI‐specific solver. This enabled us to use efficient solutions to the Bloch equations, and to implement an adaptive time‐stepping based on information already available in the sequence, contributing to the simulation speed and accuracy.

We called our simulator “Koma,” inspired by the Japanese word for spinning top, as its physics resemble MRI's.

## 2 METHODS

In this section we start by describing in detail the simulation framework and its implementation (2.1), we then describe the experiments we did for comparison (2.2), and finally, we showcase an application in quantitative imaging (2.3).

### 2.1 The simulator

#### 2.1.1 Overview

**FIGURE 1:**
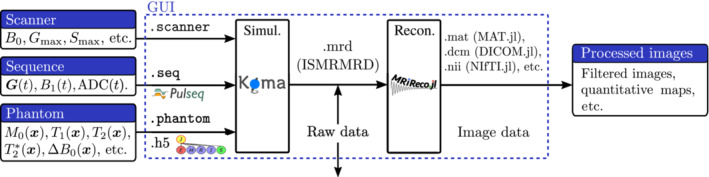
The simulation pipeline is divided in two steps: simulation, and reconstruction. Each arrow represents a file, and the pipeline can be initiated from any stage of the data workflow. Note that we are able to both read and write ISMRMRD files from the Graphical User Interface.

KomaMRI.jl has three input objects that describe the scanner, the phantom, and the sequence (Figure 1):

- Scanner: Description of the hardware specifications such as $B_{0}$, maximum gradient $G_{\max}$ and slew rate $S_{\max}$. Other properties, such as inhomogeneity of the field $\Delta B_{0}\left(r\right)$ and coil sensitivity should also be defined here (these are not available yet in the public version).
- Phantom: Representation of the virtual object with properties such as the position x of the spins, proton density $M_{0}$, $T_{1}$, $T_{2}$, $T_{2}^{*}$, off‐resonance Δω, nonrigid motion field $u\left(x,t\right)$, etc.
- Sequence: Contains the Gradient waveforms $G\left(t\right)$, radiofrequency (RF) pulses $B_{1}\left(t\right)=B_{1,x}(t)+iB_{1,y}(t)$ (where $B_{1,x}(t)$ and $B_{1,y}(t)$ are the x and y components of the RF pulse), and data acquisition timing $\text{ADC}\left(t\right)$.

We used the JLD2.jl package to implement our own HDF5‐compatible file formats, .scanner, .phantom and .seqk, to load these objects. For the sequence, the simulator also accepts the newest versions of the Pulseq format (versions 1.2‐1.4). This format gives our simulator more compatibility and convenience since it is also read by some real scanners.

The output of the simulator is written in the ISMRM Raw Data format.^28^ This allows to test the reconstruction with different sources of data, simulated or real, and also allows to link the simulator with an external reconstruction.

#### 2.1.2 Physical and mathematical background

Koma simulates the magnetization of each spin by solving the Bloch equations in the rotating frame

(1) $$\frac{dM}{dt}=\gamma M\times B+\frac{\left(M_{0}-M_{z}\right)\hat{z}}{T_{1}}-\frac{M_{x}\hat{x}+M_{y}\hat{y}}{T_{2}},$$

with γ the gyromagnetic ratio, $M={\left[M_{x},M_{y},M_{z}\right]}^{T}$ the magnetization vector, and

(2) $$B={\left[B_{1,x}\left(t\right),B_{1,y}\left(t\right),G\left(t\right)\cdot x\left(t\right)+\frac{\Delta \omega (t)}{\gamma }\right]}^{T},$$

the effective magnetic field. $M_{0}$ is the proton density, $T_{1}$ and $T_{2}$ are the relaxation times, and Δω is the off‐resonance, for each position.

The solution of Equation (1) for a single spin is independent of the state of the other spins in the system, a key feature that enables parallelization.^15^

**FIGURE 2:**
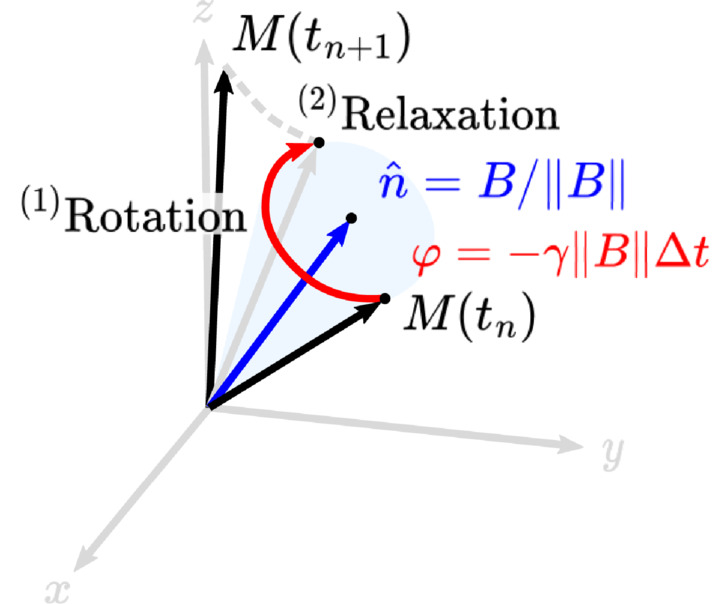
Solution of the Bloch equations for one time step can be described by (1) a rotation and (2) a relaxation step.

Our simulator uses a first‐order splitting method^29^ to simplify the solution of Equation (1). This reflects mathematically the intuition of separating the Bloch equations in a two‐step process, rotation and relaxation, for each time step $\Delta t=t_{n+1}-t_{n}$ (Figure 2). The rotation is described by

(3) $$\frac{dM^{\left(1\right)}}{dt}=\left[\begin{array}{ccc} 0 & \gamma B_{z} & -\gamma B_{y}\\ -\;\gamma B_{z} & 0 & \gamma B_{x}\\ \gamma B_{y} & -\gamma B_{x} & 0 \end{array}\right]M^{\left(1\right)},$$

with initial condition $M^{\left(1\right)}(t_{n})=M(t_{n})$, and the relaxation is described by

(4) $$\frac{dM^{\left(2\right)}}{dt}=\left[\begin{array}{ccc} -\;\frac{1}{T_{2}} & 0 & 0\\ 0 & -\frac{1}{T_{2}} & 0\\ 0 & 0 & -\frac{1}{T_{1}} \end{array}\right]M^{\left(2\right)}+\left[\begin{array}{c} 0\\ 0\\ \frac{M_{0}}{T_{1}} \end{array}\right],$$

with $M^{\left(2\right)}(t_{n})=M^{\left(1\right)}(t_{n+1})$. Then, the magnetization at the end of the time step is $M(t_{n+1})=M^{\left(2\right)}(t_{n+1})$.

Furthermore, we define two regimes in the pulse sequence: excitation and precession. During the latter, the excitation fields are nulled: $B_{x}=B_{y}=0$ in Equation (3). In the precession regime, the operator splitting method gives an exact solution, whereas during the excitation regime the method has $O\left(\Delta t^{3}\right)$ convergence.^30^

From this point forward, we will drop the vectorial notation for M and $B_{1}$, and we will use $M_{xy}=M_{x}+iM_{y}$ and $B_{1}=B_{1,x}+iB_{1,y}$ to describe the simplifications made in each regime.

The rotations during the excitation regime are stored in their spin‐domain or SU(2) representation:

(5) $$Q=\left[\begin{array}{cc} \alpha & -\beta^{*}\\ \beta & \alpha^{*} \end{array}\right],\;\text{with}\;{\left|\alpha \right|}^{2}+{\left|\beta \right|}^{2}=1,$$

characterized by the Cayley–Klein complex parameters or Spinors for short $\left(\alpha ,\beta \right)$.^31^ Spinors can represent any three‐dimensional rotation as

(6) $$\begin{array}{ll} \alpha & =\cos\left(\frac{\varphi }{2}\right)-i\;n_{z}\sin\left(\frac{\varphi }{2}\right). \end{array}$$

(7) $$\begin{array}{ll} \beta & =-i\;n_{xy}\sin\left(\frac{\varphi }{2}\right). \end{array}$$

To solve Equation (3) the parameters for the Spinors are $n_{xy}=B_{1}/\left\|B\right\|$, $n_{z}=B_{z}/\left\|B\right\|$, and

(8) $$\varphi =-\gamma \left\|B\right\|\Delta t,$$

is the phase accumulated due to B, the effective magnetic field. Then, the application of a Spinor rotation to a magnetization element is described by the operation

(9) $$\left[\begin{array}{c} M_{xy}^{+}\\ M_{z}^{+} \end{array}\right]=\left[\begin{array}{c} 2\alpha^{*}\beta M_{z}+\alpha^{*}{\;}^{2}M_{xy}-\beta {\;}^{2}M_{xy}^{*}\\ \left(\left|\alpha \right|{\;}^{2}-\left|\beta \right|{\;}^{2}\right)M_{z}-2\text{Re}\left(\alpha \beta M_{xy}^{*}\right) \end{array}\right].$$

For the precession regime, all the rotations are with respect to z, and therefore they can be described with a complex exponential applied to the transverse magnetization

(10) $$M_{xy}^{+}=M_{xy}e^{i\varphi },$$

where φ is defined in Equation (8).

Finally, to solve the relaxation step described in Equation (4) the magnetization is updated by

(11) $$\begin{array}{ll} \left[\begin{array}{c} M_{xy}^{+}\\ M_{z}^{+} \end{array}\right]=\left[\begin{array}{c} e^{-\Delta t/T_{2}}M_{xy}\\ M_{z}e^{-\Delta t/T_{1}}+M_{0}\left(1-e^{-\Delta t/T_{1}}\right) \end{array}\right]. & \end{array}$$

The presented model solves the Bloch equations for a single isochromat, and by itself cannot simulate all the physical properties of interest in MRI. Other simulators implement more general equations like Bloch–Torrey,^14^, ^32^ to simulate diffusion, and Bloch–McConnell,^19^, ^33^, ^34^ to simulate chemical exchange,^35^, ^36^ magnetization transfer^37^, ^38^ and spin‐lock effects.^39^, ^40^ Besides nonrigid motion, we assume constant magnetic properties over time, so dynamic contrast‐enhanced imaging^41^ would require an extension of the model.

Nevertheless, incorporating other realistic effects like $T_{2}^{*}$ and diffusion could be easily added by increasing the number of spins. On the other hand, the Bloch–McConnell equations could also be implemented accurately with the operator splitting method.^29^ While Koma is not as feature‐complete as other well‐established simulators, we focused on improving its speed, extensibility, and ease of use, and will keep adding more features in the future.

#### 2.1.3 Simulation blocks, regime switching, and sequence‐aware time stepping

**FIGURE 3:**
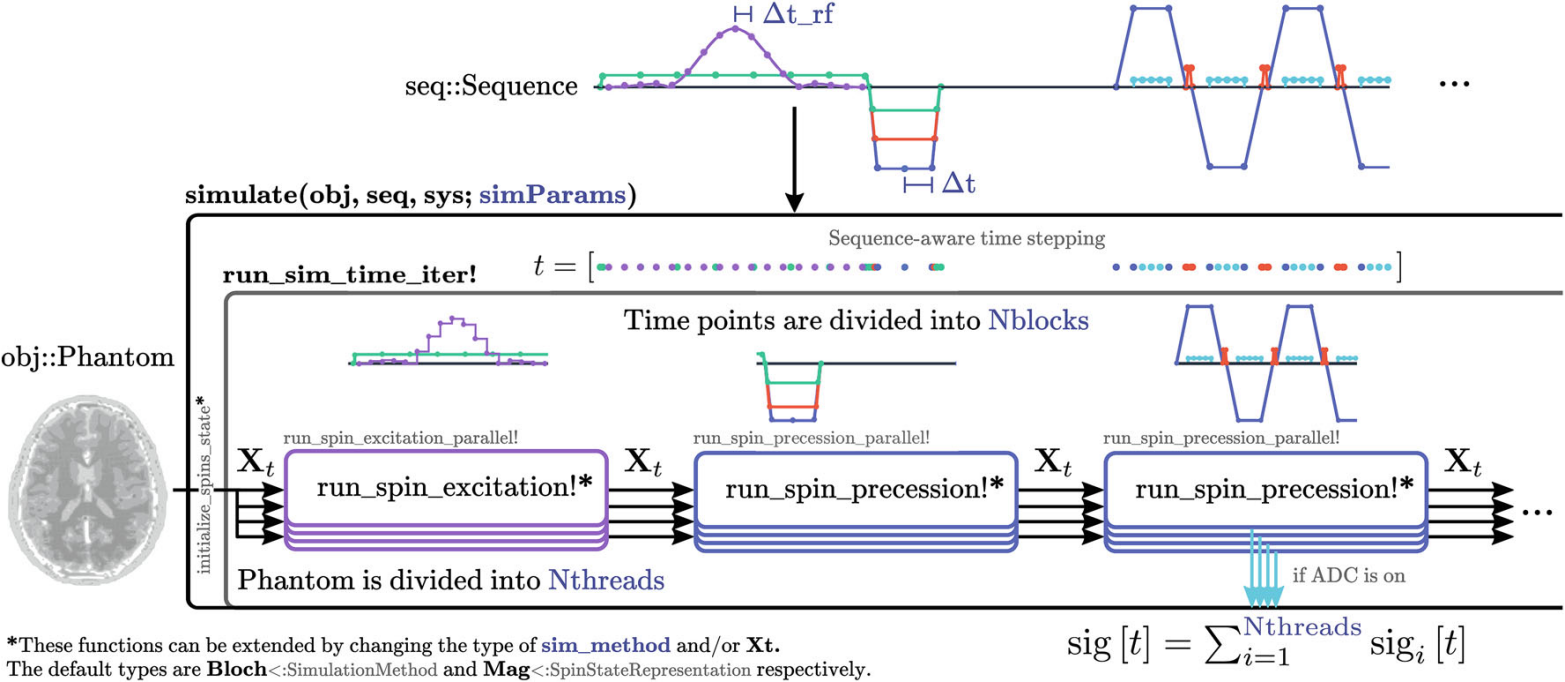
The sequence seq is discretized after calculating the required time points in the wrapper function simulate. The time points are then divided into Nblocks to reduce the amount of memory used. The phantom obj is divided into Nthreads, and Koma will use either run_spin_excitation! or run_spin_precession! depending on the regime. If an ADC object is present, the simulator will add the signal contributions of each thread to construct the acquired signal $\text{sig}\left[t\right].$ All the parameters: Nthreads, Nblocks, Δt_rf, and Δt, are passed through a dictionary called simParams as an optional parameter of the simulate function.

To reduce the memory usage of our simulator, we subdivided time into Nblocks (Figure 3). Koma classifies each block in either the excitation regime or the precession regime before the simulation.

For precession blocks, we can improve the accuracy of the simulations by using the integral representation of Equation (10), obtained by applying the limit as Δt→0 of iterated applications of Equation (10), giving a phase of

(12) $$\begin{array}{ll} \varphi & =-\gamma \int_{t_{i}}^{t_{i+1}}B_{z}(\tau )\;d\tau . \end{array}$$

(13) $$\begin{array}{ll} & =-\gamma \int_{t_{i}}^{t_{i+1}}\left(G\left(\tau \right)\cdot x\left(\tau \right)+\frac{\Delta \omega (\tau )}{\gamma }\right)\;d\tau . \end{array}$$

Assuming that during the *i*th simulation block ($t\in \left[t_{i},t_{i+1}\right]$) the gradients $G\left(t\right)$ are piece‐wise linear functions, and $x\left(t\right)$ and Δω(t) are approximately constant, then, if we use the trapezoidal rule to obtain the value of this integral, we will obtain an exact result by sampling just the vertices of $G\left(t\right)$, greatly reducing the number of points required by the simulation. We will only need intermediate points in the case of motion and for recording the sampling points as required by the Analog to Digital Converter (ADC). The user can control the time between intermediate gradient samples with the parameter Δ t (Figure 3).

We can do something similar with $B_{1}\left(t\right)$ in the excitation regime. If we assume $B_{1}\left(t\right)$ is a piece‐wise constant function (or concatenation of hard pulses), then Equation (9) will give an exact solution to Equation (3).^42^ The parameter Δ t_rf manages the time between RF samples (Figure 3).

Thus, Koma uses the rationale mentioned above to: (1) call different methods based on the regime of each block, while also (2) obtaining a variable time stepping schedule that adapts to the sequence needs. We named the latter sequence‐aware time stepping (Figure 3). While this concept is not new per se,^15^, ^19^, ^43^ in Koma we directly calculate the required simulation points from Pulseq files or a designed Sequence and then provide a convenient DiscreteSequence type to the user to simulate with our default Bloch<:SimulationMethod or to use in their custom simulation method. We comment further into this in Section 2.1.7.

#### 2.1.4 GPU/CPU parallelization

One key advantage of using Julia is its support for CPU parallelization using macros like Threads.@threads before a for loop, or the package ThreadsX.jl. Using these resources, we increased the simulation speed by separating the Bloch calculations into Nthreads. This separation is possible as all magnetization vectors are independent of one another. To ensure thread safety, we stored the acquired signals per thread in different matrices to add them later into a signal matrix sig[t] (Figure 3).

Julia also has native GPU support using the package CUDA.jl. This package supports operations using CuArray types which run as GPU kernels, but direct GPU kernel programming is also possible. To transfer variables between CPU and GPU memory, we used the packages Adapt.jl and Functors.jl. These packages let us transparently transfer our data types from CPU to GPU without losing the type abstractions. Then, the transfer looks like obj = obj |> gpu. Our data types, Phantom, DiscreteSequence, and Mag<:SpinStateRepresentation were used in this way, to then perform the simulation inside the functions run_spin_excitation! and run_spin_precession!.

It was important to ensure the type stability of our simulation functions to give enough information to the compiler to infer the concrete type of every variable, enabling high performance. Moreover, we had special care to perform in‐place operations and not generate unnecessary variable copies using the @view macro in the functions run_spin_excitation_parallel!, run_spin_precession_parallel!, and run_sim_iter!. Finally, we used NVIDIA Nsight Systems to profile GPU performance with the NVTX.@range and CUDA.@profile macros.

#### 2.1.5 Reconstruction

For the image reconstruction, we used MRIReco.jl,^23^ a reconstruction framework written in Julia with comparable performance to the Berkeley Advanced Reconstruction Toolbox (BART),^24^ a state‐of‐the‐art reconstructor. Coincidentally, as of version 2.9, JEMRIS uses BART as reconstructor. The obtained image can be saved in multiple formats such as .mat, .dcm, and others, as shown in Figure 1.

The reconstructor can also load directly the raw data from an ISMRMRD file, skipping the simulation.

#### 2.1.6 Graphical user interface

For the GUI we used Blink.jl, a framework to develop applications using web technologies. This package is a wrapper of Electron, and can serve HTML content in a local window. The communication between Julia and this web page is done using JavaScript.

**FIGURE 4:**
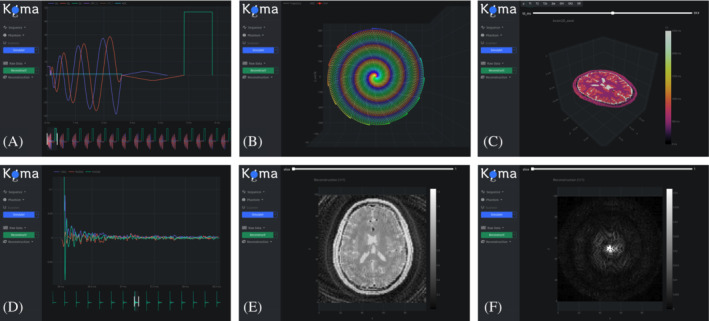
Koma's graphical user interface (GUI): (A) Sequence (interleaved spiral), (B) k‐space, (C) Phantom, (D) Raw Data, (E) Image, and (F) k‐space data (Fourier transform of (E)). The menu at the left of the GUI tries to mimic the pipeline of Figure 1, where the blue button calls Koma and the green button calls MRIReco.jl.

The GUI allows the user to easily plot the sequence, k‐space, phantom, acquired signal, and reconstructed images (Figure 4). Plots are done using the PlotlyJS.jl package, which also allows to export them to .svg files.

#### 2.1.7 Extensibility

As we mentioned in the Introduction, in Julia, functions use different methods based on the input types via multiple dispatch. We used this to specialize the simulation functions for a given sim_method<:SimulationMethod specified in simParams. For a given simulation method, the function initialize_spin_state outputs a variable Xt<:SpinStateRepresentation that is passed through the simulation (Figure 3). For the default simulation method Bloch, the spin state is of type Mag, but can be extended to a custom representation, like for example EPGs^44^ or others. Then, the functions run_spin_excitation! and run_spin_precession! can be described externally for custom types sim_method and Xt, extending Koma's functionalities without the need of modifying the source code and taking advantage of all of Koma's features.

### 2.2 Experiments

To test Koma, we performed two kinds of experiments: one to compare the quality of the results and the execution speed, and the second one to compare its usability.

#### 2.2.1 Simulation accuracy and speed

To test the accuracy of our simulator, we compared Koma with the latest version of JEMRIS (v2.9), which has been compared with real MRI acquisitions.^45^

We did two‐dimensional experiments with different number of spins to look at the scalability of the simulations. The experiments were as follows:

- Echo‐planar imaging (EPI) acquisition of a column of spins (one‐dimensional data in a two‐dimensional image). This column was subdivided into four segments of length l=50 mm. The properties of each segment were $M_{0}=[1,0.5,1,0.5]$, and $T_{1}=T_{2}=[100,50,100,50]\;\text{ms}$.
- EPI acquisition of two concentric circles (R=50 mm and r=25 mm) with a constant frequency offset for the circle in the middle (Δω=200rad/s). Both circles had the same $M_{0}=1$ but different relaxation times ($T_{1}^{r}=T_{2}^{r}=50\;\text{ms}$, and $T_{1}^{R}=T_{2}^{R}=100\;\text{ms}$).
- EPI acquisition of a human brain, with properties obtained from the BrainWeb database,^46^ including a realistic off‐resonance field (Δω's ranging from −400 to 1200 rad/s).
- EPI acquisition of the same brain of (c) without off‐resonance but with motion. We applied a displacement field in the y‐direction of $u_{y}(x,t)=v_{y}t$ with $v_{y}=0.1\;m/s$.
- Spiral acquisition of the same brain of (c) without off‐resonance.

The EPI was a single‐shot sequence with echo time (TE)=100 ms. The spiral acquisition was a single‐shot sequence with TE=0.1 ms. Both were Gradient Echo sequences with the same field of view $(\text{FOV})=230\times 230\;{\text{mm}}^{2}$ and spatial resolution/voxel size Δx=2.3mm, while the resolution of the phantoms were $\Delta x_{\text{obj}}=1\;\text{mm}$. All the images were reconstructed in a 100×100 matrix with $\text{FOV}=230\times 230\;{\text{mm}}^{2}$. Both sequences used hard RF pulses.

For the reconstruction of the spiral data for both simulators we used MRIReco.jl, and not BART for JEMRIS since it uses a different implementation of the NUFFT algorithm, and we wanted to keep the image comparison as fair as possible.

To compare the simulation accuracy, for each experiment the signals were normalized by JEMRIS' signal maximum, and then we calculated the Mean Absolute Difference (MAD) between them, $\text{MAD}\left(x,\hat{x}\right)=\frac{1}{n}\sum_{i=1}^{n}\left|x_{i}-{\hat{x}}_{i}\right|$. The differences are shown as a percentage of JEMRIS' signal maximum (*k*‐space center).

All these examples were run in a computer with an 11th Gen Intel Core i7‐1165G7 @ 2.80GHz × 8, with four physical cores, 16 GB RAM, a GPU GTX 1650 Ti (4 GiB of memory), and an eGPU RTX 2080 Ti (11 GiB of memory). For these examples, we only reported times for the faster GPU RTX 2080 Ti.

On the other hand, we compared the speed of our simulations against MRiLab, an open‐source GPU‐accelerated MRI simulator. For this, we replicated MRiLab's gradient echo multishot spiral sequence “PSD_GRE3DSpiral” (TE=50 ms, TR=10 s, and Δx=2.5mm), which contains a slice‐selective since RF pulse with a slice thickness of 6 mm, in conjunction with an eight‐shot spiral acquisition. We selected this sequence to stress test both simulators, as it has arbitrary waveforms for both RF and gradients pulses. We used the standard resolution ($\Delta x_{\text{obj}}=2\;\text{mm}$) three‐dimensional brain phantom present in MRiLab. We followed their simulation procedure and only simulated in a slab of $N_{\text{spins}}=20,630$ contained within the slice selection. We paid special attention to matching the number of time points and the spiral waveforms^47^ to get comparable results. We ran this simulation in the CPU, and both GPUs, for both simulators 20 times for each device and calculated the mean and SD.

We used the same computer as in the accuracy experiments, but we ran the test with both GPUs. We also simulated a similar sequence in JEMRIS to have as a reference.

#### 2.2.2 User experience

**FIGURE 5:**
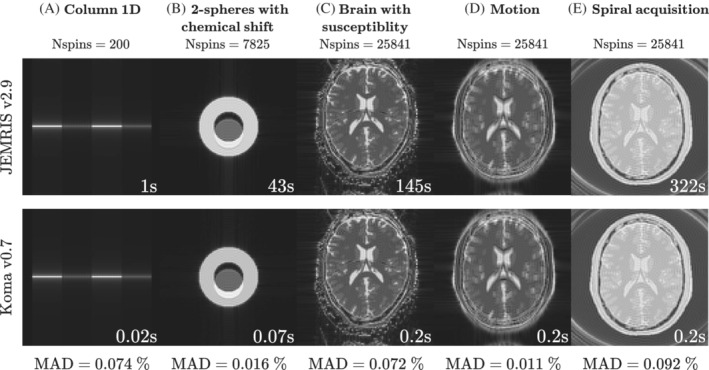
Simulations (A), (B), (C), and (D) used an echo‐planar imaging (EPI) acquisition with TE=100 ms, but (E) used a spiral acquisition with echo time (TE)=0.1 ms. All the simulations were reconstructed in a matrix of 100×100 with $\text{FOV}=230\times 230\;{\text{mm}}^{2}$. To compensate for the differences in the NUFFT reconstruction between BART and MRIReco.jl, both results in (E) used MRIReco.jl. We compared the accuracy of our simulations against JEMRIS by calculating the Mean Absolute Difference (MAD) of the normalized simulated signals.

To compare the ease of use for first‐time users, we designed a pilot experience with students of an Imaging course in Engineering, where they learned some fundamentals of MRI. The experience consisted in identifying the artifacts generated by the presence of different degrees of off‐resonance and motion (like examples (C) and (D) of Figure 5). They were to compare the artifacts of Gradient Echo and Spin Echo EPI acquisitions.

There were 19 students in the class. We divided them in two groups. The first half of the students programmed the Gradient Echo sequence and the other half the Spin Echo sequence. Each student performed six experiments per simulator with brain phantoms ($N_{\text{spins}}=5890$) with different levels of off‐resonance and motion.

For the first part of the assignment, which tested from the installation to the first simulation, they used JEMRIS v2.8.3, MRiLab 1.3, and Koma v0.3.8. For the second part, they used JEMRIS and Koma to generate the EPI sequences and different phantoms separately. We made tutorials to help them install the simulators and gave them examples of how to set up a simulation in all software packages. They used the same phantoms and sequences. For the sequence, they had to calculate the timings and gradients strengths with the information taught during the course.

To gather information about how long it took them to perform each task and their perceived level of difficulty, they filled out a form (available in Koma's GitHub) and returned it with their reports. The difficulty level of each task was rated on a Likert scale from 1 to 5, with 1 being hard and 5 being easy.

The students ran the simulations on their personal computers.

### 2.3 Magnetic resonance fingerprinting

We used our simulator to showcase its potential, simulating a quantitative MRF acquisition.^3^

The sequence was a radial balanced steady state free precession (bSSFP) sequence ($N_{\text{spokes}}=158$) to acquire the signal fingerprints for each pixel. The MRF sequence had an initial inversion pulse with TI of 50 ms. For the first 500 TRs of the sequence, we used a Perlin‐noise^48^ flip angle pattern, and for the last 500 TRs, we used a noisy sinusoid flip angle pattern between 0 deg and 80 deg similar to Ma.^3^ The TRs were randomly distributed between 14.5 and 18.0 ms, and a constant TE of 5 ms was used. A dictionary was generated to do the fingerprint matching with the following ranges of $T_{1}$ and $T_{2}$: $T_{1}$ (300–2500 ms, every 10 ms) and $T_{2}$ (40–350ms, every 4 ms).

The Phantom object was a two‐dimensional axial brain constructed using the BrainWeb database^46^ with 6506 spins. Two variations of the sequence were tested, by rotating the spokes uniformly ($\Delta \theta =\pi /N_{\text{spokes}}$) and by the tiny golden angle (Δθ=π/(ϕ+6), with ϕ the golden ratio).^49^

The tissue property maps were obtained by performing an external reconstruction. The methods used were one of the following:

- Full‐Dict: Filtered back‐projection reconstruction for each TR, and then selected the closest dictionary entry by using the maximum dot product.
- LRTV: Low‐rank dictionary matching with total variation regularization,^50^, ^51^, ^52^ with a dictionary of a reduced rank of 5.

Finally, we compared the quantitative maps on white (WM) and gray matter (GM) regions by using the Mean Absolute Error.

This simulation was run on the same computer as the one used in Section 2.2.1.

## 3 RESULTS

In this section, we report the results obtained from the experiments that compare Koma against other open‐source MRI simulators, from the usability tests, and from the MRF showcase.

### 3.1 Simulation accuracy and speed

For the simulated examples described in Section 2.2.1, we obtained accurate results with MADs below 0.1% when compared to JEMRIS (Figure 5) and the simulation times were 50, 614, 725, 725, and 1610 times faster, respectively, for Koma. In these tests, Koma improves the simulation time considerably when the complexity of the problem is increased.

When we tested the simulation speed against MRiLab (Table 2), we found that we had slower CPU performance, but we were 2.6 times faster for the GTX 1650Ti and 6.0 times for the RTX 2080 Ti. We think the CPU results show that we still perform unwanted synchronizations between threads, a problem that our GPU implementation would not suffer as we use Nthreads=1 by default. An interesting result was that MRiLab was slower for the more powerful RTX 2080 Ti. This is probably explained by CPU‐to‐GPU memory transfers as the external GPU could be bottle‐necked by the Thunderbolt bandwidth capacity. We put most of our attention on the GPU performance, specifically to reduce the number of memory transfers to the GPU by profiling with NVIDIA Nsight tools, which are easily accessed within Julia.

**TABLE 2:** Simulation times for CPU, and GPU acceleration.

	CPU	GPU	
Name	Intel i7‐1165G7	GTX 1650 Ti	RTX 2080 Ti
JEMRIS	≈7 min	‐	‐
MRiLab	**1.56** **s** ± **0.07** **s**	0.84 s ± 0.02 s	0.91 s ± 0.02 s
Koma	1.82 s ± 0.17 s	**0.32** **s** ± **0.02** **s**	**0.15** **s** ± **0.01** **s**

### 3.2 User experience

Students reported no problem installing Julia (mean 4.7/5), Koma (mean 4.2/5), JEMRIS (mean 3.8/5), and MRiLab (mean 4.3/5). Regarding the time taken to install each simulator, most of the students were able to install Koma (mean 13.2 min), JEMRIS (mean 33.8 min), and MRiLab (mean 16.9 min) in less than 40 min.

Their first simulation took them more time in JEMRIS (mean 19 min) and MRiLab (mean 13.9 min) than in Koma (mean 5.7 min). 31% of the students could not simulate on MRiLab (six students using Mac OS), so we decided to only use Koma and JEMRIS for the rest of the activities.

Not all students used Koma's GPU features, as only seven students (37%) had a compatible GPU. Those students experienced a slower first simulation (mean 7 min) than those without a GPU (mean 5 min). This slowdown is because the first simulation included installing the CUDA drivers and the slower compilation of the GPU version of the functions.

To program the pulse sequence, students found that JEMRIS's GUI was slightly better (mean 3.85/5) than Koma's code‐based pulse programming (mean 3.69/5). This makes sense since the students' self‐reported computational expertise was less than expected (Q1/median/Q3=1.6/2.2/2.6, where 3 meant “I can implement my ideas easily in one programming language”). This feedback helped us improve the pulse sequence programming by implementing our Pulseq file reader, which enables programming the sequence in JEMRIS's GUI.

Nevertheless, the students also commented that it was unintuitive that the gradients' strengths in the JEMRIS's GUI were not in mT/m but scaled by γ, so $G_{\text{JEMRIS}}=\gamma_{\mathrm{rad}/\mathrm{us}/\mathrm{mT}}\cdot G_{\mathrm{mT}/m}\approx 0.267538\cdot G_{\mathrm{mT}/m}$. This caused many failed simulations, which prompted us to do an additional tutorial session.

They also readily modify the phantoms with different levels of off‐resonance and motion with JEMRIS (mean 4.27/5) and with Koma (mean 4.24/5).

**FIGURE 6:**
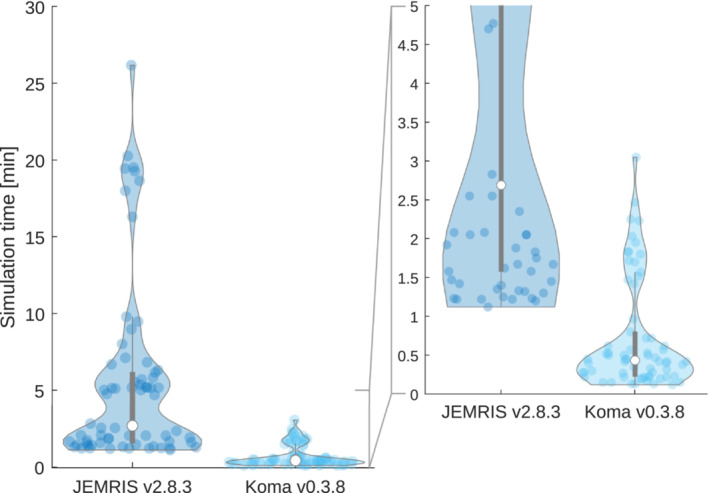
The students' simulation times with their PCs. Each dot represents one of six experiments they needed to make. We only show the results for the 11 students that successfully simulated all the examples.

Finally, their reported median simulation speeds were 8.4 times faster with Koma than with JEMRIS (Figure 6), and 65% ended up recommending Koma over JEMRIS.

### 3.3 Magnetic resonance fingerprinting

**FIGURE 7:**
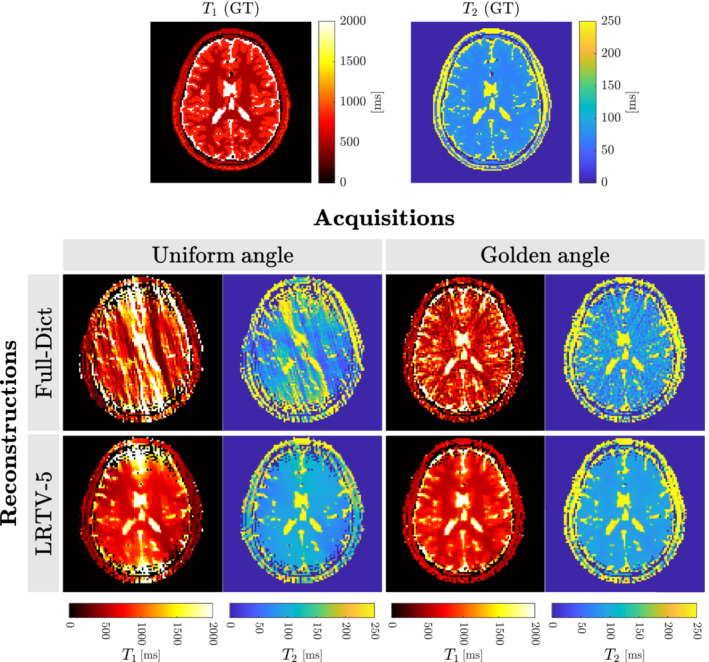
Comparison of different MRF acquisitions and reconstruction methods. (GT) Ground‐truth, (Full‐Dict) reconstruction using the full dictionary, and (LRTV‐5) Low‐rank with Total Variation reconstruction with rank of 5 for the dictionary.

The MRF simulation took approximately 20 s to finish. Results of using different sequences and reconstruction algorithms for our MRF simulated acquisition are shown in Figure 7. The best overall pipeline was to rotate the spokes by the tiny golden angle and then reconstruct using LRTV, which had the lowest mean absolute error's of (WM‐T1, GM‐T1, WM‐T2, GM‐T2) = (55, 26, 15, 13) ms. Thus, we reproduced results from the state‐of‐the‐art in MRF acquisition just by using simulations.^49^, ^51^

This showed the flexibility of our simulator to test novel sequences. Ideas proven in simulation could potentially be directly executed on a Pulseq‐compatible scanner.^25^

## 4 DISCUSSION

### 4.1 Efficiency

Similarly to other MRI simulators,^15^, ^19^, ^43^ Koma's variable time‐stepping accelerates the simulations, nevertheless, this is not the only contributing factor to efficiency. Another factor is that Koma chooses a different simulation method depending on the sequence regime (excitation or precession). For RF blocks, the method used rotates the magnetization assuming a constant effective field for each time step. For the rest of the sequence (gradient‐only or free precession blocks) we assumed a linear effective field for each time step and used trapezoidal integration to estimate the accumulated phase. These methods offer accurate solutions given that we chose the time points carefully, and also that we approximately satisfy the assumptions described in Section 2.1.3, which is generally the case in MRI (even with motion, see Figure 5). Thus, prior knowledge of the MRI physics and the expected characteristics of $B_{1}$ and G(t) provides insight to choose a less computationally intensive method needed for an accurate simulation. This was seen in the students experience (3.2) where they used a previous version of the software that had uniform time steps. Even then, Koma's MRI‐specific solver outperformed the CVODE general ODE solver. This result is not explained by the use of GPUs, as most of the students did not have one in their personal computers.

In summary, the speed acceleration in our simulator comes from the time‐stepping procedure, from the switching between regimes, and from the CPU parallelization and GPU implementation. But there is still room for growth, we can still improve our CPU performance (as shown in Table 2), and we can do the same for the GPU implementation. Currently, we are just performing the operations with CuArray types. A proper implementation of some of the functions as GPU kernels, which is also possible in Julia, will potentially further accelerate our simulations. For example, the method to simulate RF‐blocks with long soft RF pulses (like adiabatic pulses) is not as efficient as one would expect. The code has already been set up for implementing GPU kernels by using functions that perform in‐place operations without returning a result.

### 4.2 Open source and community standards

Our simulator is open‐source, and it is already available on GitHub (github.com/cncastillo/KomaMRI.jl). Furthermore, we seek contributions from the community. For this, we are currently developing documentation with examples using and extending Koma's functionalities. We made and continue to make an effort to make Koma as modular as possible to facilitate its modifications.

We used community‐driven and public file formats to increase reproducibility. Koma writes and reads ISMRM raw data, making it compatible with other reconstruction software. Koma's GUI can display and reconstruct raw data acquired on an actual scanner using MRIReco.jl.

We can also read the newest versions of the Pulseq standard, enabling the generation of the sequence directly in Koma, in MATLAB's Pulseq toolbox, or by using JEMRIS' GUI. If these standards change, the file readers could easily be updated. Our simulator is one of the first to receive Pulseq files as the sequence definition. This will allow us to customize pulse sequences and then test them in real scanners.^53^

The use of MRIReco.jl brings state‐of‐the‐art reconstructions to Koma. This reconstructor can do direct or regularized iterative reconstructions (FISTA,^54^ ADMM,^55^ and others). This software also brings flexible and easily customizable reconstructions (see MRIReco.jl documentation).

Similarly to MRIReco.jl, we used a dictionary (Dict) to store all the simulation parameters, which can be easily updated to add new parameters. All these parameters are saved to the raw data for later inspection.

### 4.3 Maintainability and reproducibility

While performing the experiments for this work, we experienced problems running JEMRIS and MRiLab in modern systems. For JEMRIS, we could not perform multithreaded simulations out of the box, as some library versions are not supported anymore. We had to compile the package and change the source code to fix some problems with up‐to‐date versions of CVODE. On the other hand, for MRiLab, the GPU simulations had a similar problem, as it assumed that the system had an old version of CUDA. We had to fix the source code and makefiles to compile for modern versions of CUDA and the MEX libraries. Despite this, we were not able to recover all the functionalities, like the ability to export the signal as ISMRMRD, as it uses a deprecated version of the library.

We believe this experience perfectly represents a common problem for the MR community: maintainability and reproducibility of the software we produce. While not perfect, we believe that Julia helps in minimizing many of these problems. Its modular approach incentives the separation of packages with specific functionality, which are easier to maintain. Furthermore, all Julia packages are associated with a GitHub page, and to be registered, each new package version is required to maintain or improve the Code Coverage of the tests, and pass the Continuous Integration which assesses the package in multiple versions of Julia and operating systems: Windows, Linux, and Mac. Moreover, even if a package is no longer maintained by the creator, if shared, the Manifest.toml of a package contains all the specific versions of each module, and the environment can be replicated by using the command Pkg.activate(“.”), enabling reproducibility. We shared not only the Manifest.toml, but all the code used to replicate the simulations presented in this work in Koma's GitHub.

Julia not only brings high‐performance and easy‐to‐read code but also forces package developers to produce professional software, decreasing the technical debt passed to new researchers.

### 4.4 Limitations

The current implementation of Koma suffers from the same limitations as other Bloch simulators,^19^ which means that some intra‐voxel effects, like $T_{2}^{*}$ and diffusion, require many spins per voxel, which in turn affects the simulation speed.

An important issue with Bloch simulators is the potential aliasing or spurious echoes when simulating gradient spoilers. They arise due to the finite separation between spins (discrete delta functions) which produces overlapping or aliasing in the Fourier domain. The simplest solution, as before, is to increase the number of spins at the cost of extra computational load. To solve this problem, alternative intermediate solutions should be explored. One of them is to use a different model to describe the spins' state as the one used by the hybrid Bloch‐EPG.^56^

While it is straightforward to implement, we do not yet have multiple coils in our simulator. The lack of coils precludes its ability to simulate conditions at ultra‐high fields where coil combination is an issue,^57^ or in highly accelerated sequences where the coil noise characteristics are essential, like in wave‐CAIPI.^58^ Also, our simulator is not currently considering some effects, including eddy currents, concomitant gradients, temperature changes, or the drift on the k‐space center produced by long readouts.^59^

We designed our simulator to run reasonably fast on a student notebook. More testing is required for more complex scenarios in more powerful servers. Our software has not yet been tested in a multi‐GPU system like in Xanthis et al.,^60^ and more work is needed to take advantage of multiple GPUs.

## 5 CONCLUSIONS

In this work, we presented a new general MRI simulator programmed in Julia. This simulator is fast, easy to use, extensible, open‐source, and cross‐platform. These characteristics were achieved by choosing the appropriate technologies to write easy‐to‐understand and fast code with a flexible GUI. Furthermore, our simulation method exploits MRI physics and information about the sequence to reduce the simulation times.

We compared the accuracy of our simulations against JEMRIS, in which we showed high accuracy with MADs below 0.1%. We also compared the performance against MRiLab, showing slower CPU times but GPU performance as much as six times faster using an RTX 2080 Ti eGPU, and 2.6 times faster using a GTX 1650 Ti.

We also tested the ease of use of Koma with students without previous knowledge of MRI. Their feedback helped us improve Koma by adding compatibility with community‐driven standards like Pulseq and the ability to load JEMRIS phantoms. Thus, Koma can use the same sequences and phantoms utilized in JEMRIS. We can also receive simulations from JEMRIS, or scanner‐generated raw data, and reconstruct them in our GUI using the exported ISMRMRD file. Moreover, we can export our raw data to the same format and reconstruct the images externally.

Finally, we showcase the potential to quickly test novel pulse sequences for quantitative MRI before implementing them in the scanner by simulating different MRF acquisitions.

## Data Availability

Our simulator is open‐source, and is available on GitHub (github.com/cncastillo/KomaMRI.jl).
